# Prediction of Glucose Metabolism Disorder Risk Using a Machine Learning Algorithm: Pilot Study

**DOI:** 10.2196/10212

**Published:** 2018-11-26

**Authors:** Katsutoshi Maeta, Yu Nishiyama, Kazutoshi Fujibayashi, Toshiaki Gunji, Noriko Sasabe, Kimiko Iijima, Toshio Naito

**Affiliations:** 1 Faculty of Informatics and Engineering The University of Electro-Communications Tokyo Japan; 2 Department of General Medicine School of Medicine Juntendo University Tokyo Japan; 3 Center for Preventive Medicine NTT Medical Center Tokyo Tokyo Japan

**Keywords:** diabetes, machine learning, 75-g oral glucose tolerance test, XGBoost

## Abstract

**Background:**

A 75-g oral glucose tolerance test (OGTT) provides important information about glucose metabolism, although the test is expensive and invasive. Complete OGTT information, such as 1-hour and 2-hour postloading plasma glucose and immunoreactive insulin levels, may be useful for predicting the future risk of diabetes or glucose metabolism disorders (GMD), which includes both diabetes and prediabetes.

**Objective:**

We trained several classification models for predicting the risk of developing diabetes or GMD using data from thousands of OGTTs and a machine learning technique (XGBoost). The receiver operating characteristic (ROC) curves and their area under the curve (AUC) values for the trained classification models are reported, along with the sensitivity and specificity determined by the cutoff values of the Youden index. We compared the performance of the machine learning techniques with logistic regressions (LR), which are traditionally used in medical research studies.

**Methods:**

Data were collected from subjects who underwent multiple OGTTs during comprehensive check-up medical examinations conducted at a single facility in Tokyo, Japan, from May 2006 to April 2017. For each examination, a subject was diagnosed with diabetes or prediabetes according to the American Diabetes Association guidelines. Given the data, 2 studies were conducted: predicting the risk of developing diabetes (study 1) or GMD (study 2). For each study, to apply supervised machine learning methods, the required label data was prepared. If a subject was diagnosed with diabetes or GMD at least once during the period, then that subject’s data obtained in previous trials were classified into the risk group (y=1). After data processing, 13,581 and 6760 OGTTs were analyzed for study 1 and study 2, respectively. For each study, a randomly chosen subset representing 80% of the data was used for training 9 classification models and the remaining 20% was used for evaluating the models. Three classification models, A to C, used XGBoost with various input variables, some including OGTT data. The other 6 classification models, D to I, used LR for comparison.

**Results:**

For study 1, the AUC values ranged from 0.78 to 0.93. For study 2, the AUC values ranged from 0.63 to 0.78. The machine learning approach using XGBoost showed better performance compared with traditional LR methods. The AUC values increased when the full OGTT variables were included. In our analysis using a particular setting of input variables, XGBoost showed that the OGTT variables were more important than fasting plasma glucose or glycated hemoglobin.

**Conclusions:**

A machine learning approach, XGBoost, showed better prediction accuracy compared with LR, suggesting that advanced machine learning methods are useful for detecting the early signs of diabetes or GMD. The prediction accuracy increased when all OGTT variables were added. This indicates that complete OGTT information is important for predicting the future risk of diabetes and GMD accurately.

## Introduction

The incidence of diabetes has been increasing for the last decade and is expected to continue to increase in the future [1-3]. At present, diabetes is diagnosed and predicted based on fasting plasma glucose (FPG), glycated hemoglobin (HbA_1c_), and plasma glucose levels 2 hours after a 75-g oral glucose tolerance test (OGTT) [4]. In an OGTT, a patient is asked to ingest a glucose drink, and their plasma glucose (PG) levels and immunoreactive insulin (IRI) levels are measured before and at intervals after the glucose drink is consumed. Although OGTT provides important information regarding pathological conditions of glucose metabolism, many diabetes survey tools predict the risk of diabetes development based only on noninvasive information, such as self-administered questionnaires [5]. The combination of parameters used to diagnose diabetes helps to identify individuals with a high risk of developing diabetes in the future. Heianza et al [6] showed that the combination of HbA_1c_ and FPG is useful for finding patients with a high risk of developing diabetes. Fujibayashi et al [7] used HbA_1c_ values, FPG levels, and 2-hour PG to predict instances of high future risk of developing diabetes. Complete data, including 1-hour and 2-hour PG and IRI values obtained by OGTT, may improve the prediction accuracy for diabetes risk.

Previously, logistic regression (LR) analyses were used as initial screening tests [5,8-10]. Recently, studies have demonstrated new methods, including machine learning algorithms, big data mining approaches, and genomic information, for the improved screening and prediction of diabetes [11,12]. Machine learning methods using all relevant information from OGTTs may be able to more accurately predict the risk of developing diabetes and prediabetes. The goal of this study was to verify this hypothesis. To our knowledge, no previous study has predicted the development of diabetes using all of the information from OGTTs combined with machine learning.

We used XGBoost [13,14] for machine learning, an advanced algorithm known for obtaining the winning solutions in data competitions such as Kaggle. In addition, XGBoost has been applied to other medical fields [15-17]. Gao et al [15] compared model-based approaches (such as LRs) and model-free approaches (including using XGBoost) for the task of forecasting falls by patients with Parkinson disease. The authors reported that the model-free approach provided more reliable forecasting. Nishio et al [16] applied XGBoost and support vector machine methods to the computer-aided diagnosis of lung nodules. The authors reported that XGBoost was generally superior to support vector machine methods. Qiao et al [17] applied XGBoost and recurrent neural networks to a task of emergency room visit prediction. The authors reported that the nonlinear models had better performance than linear models.

## Methods

### Ethics Statement

This study was conducted using data from comprehensive periodic medical examinations at the Center for Preventive Medicine, NTT Medical Center Tokyo, from May 2006 to April 2017. In Japan, employers are required by the Industrial Safety and Health Law to commission medical examinations once a year to ensure the health of their employees. The Center for Preventive Medicine has been contracted by a telecommunications company, Nippon Telegraph and Telephone Corporation (NTT), to provide periodic medical examinations to their employees to comply with this law. This program involves comprehensive periodic medical examinations as well as many services beyond those mandated by law. The data used in this study were collected as part of this general health check-up program at the center. We retrieved subject clinical data from an institutional database, although the examinations were not specifically intended to collect new data for our study. Our research plan was announced on the websites of both our facility and the Center for Preventive Medicine. All subjects were informed that the clinical data obtained by the program would be retrospectively analyzed and published. In addition, it was announced that subjects could withdraw from our research study at any time. The study protocol was approved by the ethical review board of Juntendo University (No. 2017114) and the institutional ethics committee at the Center for Preventive Medicine (No. 17-664).

### Study Population

Most of the study subjects were volunteers from among the employees of NTT and their families. They were primarily healthy office workers ranging in age from 40 to 60 years, with more male subjects than females. Our investigation focused on subjects who underwent a 75-g OGTT at the center between May 2006 to April 2017. Subjects without serious diabetes or advanced renal failure were assessed regarding the status of their glucose metabolism using the OGTT.

A total of 20,458 OGTT trials were collected from 9906 subjects during the period at the center. Table 1 shows the distribution of subjects with the number of OGTT trials obtained for each during the period. Overall, 6437 subjects underwent OGTT only once, while 1 subject had 12 OGTTs.

**Table 1:** Distribution of subjects according to the number of oral glucose tolerance test trials.

Trials undergone, n	Subjects, n
1	6437
2	1157
3	736
4	459
5	331
6	251
7	172
8	143
9	93
10	81
11	45
12	1

### Data Collection

The examinations were performed on 2 consecutive days. On the first day, each patient’s weight and height were measured after the removal of shoes and heavy clothing, and blood pressure was measured with an automatic monitor with the person in the sitting position. In addition, serum samples were collected from each participant after overnight fasting and immediately subjected to biochemical analysis. The blood samples were also used to determine each subject’s HbA_1c_ level, which was measured using high-performance liquid chromatography with an automatic analyzer. On the second day, the subjects underwent an OGTT. We obtained the subjects’ FPG levels along with 1-hour and 2-hour postloading PG IRI levels during the OGTT. The Japan Diabetes Society (JDS) HbA_1c_ values were converted to National Glycohemoglobin Standardization Program values using the formula developed by the JDS [18]: HbA_1c_=[HbA_1c_(JDS)(%)×1.02+0.25(%)]. Insulin sensitivity was calculated with the insulin sensitivity index (ISI; composite) [19,20]: ISI (composite) =[10,000/ sqrt(FPG level(mg/dL)×fasting IRI level(μU/mL)×2-hour PG level(mg/dl)×2-hour IRI level(μU/mL))]. The sum of plasma glucose (SPG) is defined as SPG=FPG levels+1-hour PG level+2-hour PG level. The sum of immunoreactive insulin (S-IRI) is defined as S-IRI=fasting IRI level+1-hour IRI level+2-hour IRI level.

We defined diabetes, normal glucose tolerance (NGT), and prediabetes according to the American Diabetes Association guidelines [4]. Diabetes is defined as subjects with an FPG level ≥126 mg/dL, a 2-hour postloading PG level ≥200 mg/dL, or an HbA_1c_ concentration ≥6.5%. NGT is defined as subjects with an FPG level <100 mg/dL, a 2-hour postloading PG level <140 mg/dL, and an HbA_1c_ level <5.7%. Prediabetes is defined as subjects without diabetes who failed to have NGT. In our study, we defined glucose metabolism disorders (GMD) as either diabetes or prediabetes.

### Data Handling

#### Inclusion and Exclusion Flow

**Figure 1:**
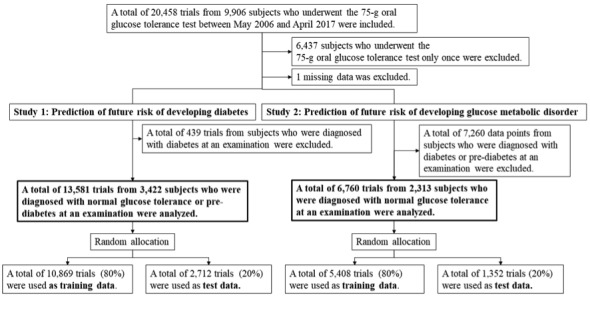
Inclusion and exclusion criteria.

Initially, a total of 20,458 OGTT trials across all subjects were included. Data were removed based on the inclusion and exclusion criteria as shown in Figure 1. First, 6437 subjects who underwent OGTT only once during the period were excluded to increase the reliability of the data. Second, missing data were excluded, and the remaining number of trials was 14,020. Then, 2 studies were conducted: predicting the future risk of developing diabetes (study 1) or GMD (study 2).

#### Study 1. Prediction of Future Risk of Developing Diabetes

Study 1 was aimed at predicting the future risk of developing diabetes. To apply supervised machine learning to the data, data labels (at risk: y=1, not at risk: y=0) were required for each OGTT trial. It is widely known that diabetes can recur even after remission. In addition, women with a history of gestational diabetes have a high risk of developing diabetes in the future [21]. We considered that subjects with a diagnosis of diabetes in the past had a high risk of developing diabetes in the future. Because of this hypothesis, we defined the risk group as follows: a subject was in the risk group (y=1) for diabetes if he or she was diagnosed with diabetes at least once during the period. We defined a subject to be in the nonrisk group (y=0) if he or she did not belong to the risk group. From 14,020 trials, 439 data points from patients diagnosed with diabetes were excluded to focus only on nondiabetic subjects.

**Figure 2:**
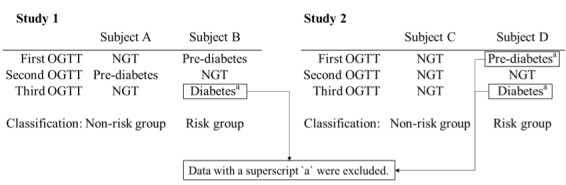
Examples of risk group and nonrisk group classifications. OGTT: oral glucose tolerance test, NGT: normal glucose tolerance.

Examples of the risk group and nonrisk group for diabetes are shown in Figure 2 (left). Subjects A and B underwent OGTT 3 times. Subject B was diagnosed with prediabetes, NGT, and diabetes in the first, second, and third OGTTs, respectively. Thus, Subject B was classified into the risk group, since he or she was diagnosed with diabetes at least once during the period. The third OGTT data point, which occurred after the diagnosis (marked with superscript a), is removed to focus only on nondiabetic data. Subject A was diagnosed with NGT, prediabetes, and NGT in the first, second, and third OGTTs, respectively. Subject A was classified into the nonrisk group, since he or she was never diagnosed with diabetes during the period.

At the end, we had a total of 13,581 OGTT trials of patients who were diagnosed with NGT or prediabetes, each of which was labeled with future risk information (y=0 or y=1). We randomly selected 10,869 (80%) for the training data and used the remaining 2712 (20%) for test data. A classification model was trained using the training data, and the prediction accuracy was evaluated with the test data. Nine classification models were trained, as described below. Table 2 shows a summary of the analyzed OGTT data. No significant differences were observed between the training and test data.

#### Study 2. Prediction of Future Risk of Glucose Metabolism Disorders

Study 2 was aimed at predicting the future risk of developing GMD, which includes either diabetes or prediabetes. Similar to study 1, we defined a subject as being in the risk group (y=1) for GMD if he or she was diagnosed with GMD (prediabetes or diabetes) at least once during the period. We defined a subject to be in the nonrisk group (y=0) if he or she did not belong to the risk group for GMD. From 14,020 trials, 7260 data points from patients diagnosed with GMD were excluded to focus only on NGT subjects.

**Table 2:** Statistical summary of the analyzed oral glucose tolerance test data (study 1).

Data points	Training data (n=10,869)	Test data (n=2712)	*P* value^a^
Age, years, mean (SD)	49.78 (9.27)	49.71 (9.16)	.71
Sex, male, n (%)	9998 (91.99)	2509 (92.51)	.61
Body mass index (kg/m^2^), mean (SD)	23.43 (3.00)	23.35 (2.98)	.21
Glycated hemoglobin (%), mean (SD)	5.50 (0.32)	5.49 (0.31)	.21
FPG^b^ (mg/dL), mean (SD)	96.59 (8.31)	96.47 (8.35)	.50
1-hour PG (mg/dL), mean (SD)	145.82 (40.67)	146.91 (40.55)	.21
2-hour PG (mg/dL), mean (SD)	111.07 (26.62)	111.26 (26.32)	.73
SPG^c^ (mg/dL), mean (SD)	353.48 (63.99)	354.63 (63.51)	.40
Fasting IRI^d^ (IU/mL), mean (SD)	6.41 (3.65)	6.40 (3.44)	.89
1-hour IRI (IU/mL), mean (SD)	55.50 (37.16)	55.04 (35.21)	.55
2-hour IRI (IU/mL), mean (SD)	41.95 (32.59)	41.32 (31.01)	.34
S-IRI^e^ (IU/mL), mean (SD)	103.87 (64.95)	102.76 (61.65)	.41
ISI^f^ (composite), mean (SD)	8.39 (5.27)	8.34 (4.89)	.59
Systolic BP^g^ (mm Hg), mean (SD)	125.38 (17.65)	125.68 (17.37)	.42
Diastolic BP (mm Hg), mean (SD)	79.74 (11.22)	80.13 (11.21)	.10
Total cholesterol (mg/dL), mean (SD)	200.80 (30.95)	200.17 (31.01)	.35
HDLC^h^ (mg/dL), mean (SD)	58.27 (14.39)	58.10 (14.45)	.59
LDLC^i^ (mg/dL), mean (SD)	118.37 (28.12)	117.83 (28.40)	.38
Triglyceride (mg/dL), mean (SD)	115.07 (79.98)	114.07 (73.87)	.54
Uric acid (mg/dL), mean (SD)	6.20 (1.27)	6.17 (1.30)	.24
UN^j^ (mg/dL), mean (SD)	13.70 (3.18)	13.57 (3.14)	.05
Serum creatinine (mg/dL), mean (SD)	0.88 (0.14)	0.88 (0.15)	.29
GOT^k^ (IU/L), mean (SD)	24.02 (9.24)	23.81 (9.28)	.29
GPT^l^ (IU/L), mean (SD)	25.56 (16.44)	24.95 (15.43)	.07
γ‐GTP^m^ (IU/L), mean (SD)	47.05 (53.13)	46.55 (48.97)	.64
Serum albumin (g/dL), mean (SD)	4.51 (0.26)	4.50 (0.26)	.11

^a^Used *t* test or chi-square test.

^b^FPG: fasting plasma glucose.

^c^SPG: sum of plasma glucose.

^d^IRI: immunoreactive insulin.

^e^S-IRI: sum of immunoreactive insulin.

^f^ISI: insulin sensitivity index.

^g^BP: blood pressure.

^h^HDLC: high-density lipoprotein cholesterol.

^i^LDLC: low-density lipoprotein cholesterol.

^j^UN: serum urea nitrogen.

^k^GOT: serum glutamic oxaloacetic transaminase.

^l^GPT: serum glutamic pyruvic transaminase.

^m^γ‐GTP: serum γ-glutamyl transpeptidase.

Figure 2 (right) shows examples of the risk group and nonrisk group for GMD. Subjects C and D underwent OGTT 3 times. Subject D was diagnosed with prediabetes, NGT, and diabetes in the first, second, and third OGTTs, respectively. Subject D was classified into the risk group of GMD, since he or she was diagnosed with prediabetes at least once during the period. The first and third OGTT, which were diagnosed with prediabetes and diabetes, respectively, were removed to focus only on NGT data. Subject C was diagnosed with NGT all 3 times. Therefore, subject C was classified into the nonrisk group, since he or she was never diagnosed with prediabetes or diabetes during the period.

Finally, we had 6760 OGTT trials of patients who were diagnosed with NGT, each of which was labeled with future risk information (y=0 or y=1). We randomly selected 5408 (80%) for training data, and used the remaining 1352 (20%) for test data. A classification model was trained using the training data, and the prediction accuracy was evaluated using the test data. Nine models were trained, as detailed in the Classification Models section. Table 3 shows a summary of the analyzed data. No significant differences were observed between the training and test data.

### Statistical Analysis

#### XGBoost

XGBoost [13] is open-source software [14] that provides a machine learning method of regression and classification using ensemble learning with gradient tree boosting (GTB) [22]. XGBoost is well known for obtaining the winning solutions in data competitions. Chen and Guestrin [13] reported that “Among the 29 challenge-winning solutions published on Kaggle’s blog during 2015, 17 winning solutions used XGBoost.” Applications of XGBoost include practical tasks such as “store sales prediction, high energy physics event classification, Web text classification, customer behavior prediction, motion detection, ad click-through rate prediction, malware classification, product categorization, hazard risk prediction, and massive on-line course dropout rate prediction.” See Chen and Guestrin [13] for details of the applications. In addition, XGBoost has been applied to medical fields [15-17].

XGBoost (or GTB) learns a regression and classification function in the data space by sequentially optimizing weak learners, called regression trees. The parameters of a regression tree consist of the tree structures and the weights of the leaf nodes. They are sequentially optimized to minimize an objective function, consisting of a fitting loss term plus a regularization term, using gradient methods. XGBoost software is designed to increase the scalability and acceleration of optimized computation for practical use. See Chen and Guestrin [13] for technical details. The underlying GTB algorithm is briefly discussed in Multimedia Appendix 1. XGBoost includes several hyperparameters—including the maximum depth of regression trees, number of weak learners, learning rate, and regularization parameters—which need to be tuned.

#### Classification Models

To predict the future risk of diabetes (study 1) or GMD (study 2), we developed 9 classification models (A to I) with different input variables, shown in Table 4.

Models A, B, and C used XGBoost. For comparison, models D to I used LR, which is traditionally used in medical research studies. For each classification model, the input variables were set as follows. Model A inputs some basic variables relevant to diabetes or GMD, without OGTT variables. Model B inputs OGTT variables (1-hour PG, 1-hour IRI, 2-hour PG, and 2-hour IRI), as well as the variables of model A. Model C inputs all the measured variables. Blood pressure, lipid parameters, uric acid values, markers of liver function, and markers of kidney function are parameters related with metabolic syndrome, fatty liver, and chronic kidney disease. These conditions are associated with diabetes and were included as variables [23-25]. Models D to F served as baselines using the well-known biomarkers FPG and HbA_1c_. Models G to I used the same variables as models A to C to directly compare the performances of XGBoost and LR.

#### Evaluation

To evaluate the 9 trained classification models, we used the receiver operating characteristic (ROC) curves and their area under the curve (AUC) values computed from the test data [26,27]. ROC curves have commonly been used in diabetes prediction research. In addition, we report the sensitivity and specificity at the cutoff values determined by the Youden index.

#### Hyperparameter Tuning of XGBoost

As mentioned, XGBoost includes several hyperparameters such as maximum depth of the regression trees, number of weak learners, learning rate, and regularization parameters that need to be tuned.

We tuned the parameters using a grid search to maximize the mean AUC value computed from 5-fold cross validation on the training data. Specifically, the training data were divided into 5 subsets at random: 4 subsets were used for training XGBoost and the other subset was used for validation. The ROC curve and AUC value can be evaluated from the validation subset. This procedure was repeated 5 times with different validation subsets. The mean AUC value can be computed by averaging the 5 AUC values. We tuned the hyperparameters, including the regularization parameters, with a grid search method to maximize the mean AUC value. After finding the optimal values of the hyperparameters, XGBoost was trained using the entire training data set. The final ROC and AUC values were then evaluated with the test data.

Given a ROC curve, a cutoff value is required to compute the sensitivity and specificity. The cutoff value was determined by averaging 5 cutoff values computed from the Youden index from 5-fold cross validation.

**Table 3:** Statistical summary of analyzed oral glucose tolerance test data (study 2).

Data points	Training data (n=5408)	Test data (n=1352)	*P* value^a^
Age, years, mean (SD)	47.13 (8.75)	46.93 (8.70)	.43
Sex, male, n (%)	4853 (89.74)	1210 (89.50)	.83
Body mass index (kg/m^2^), mean (SD)	22.71 (2.70)	22.72 (2.70)	.89
Glycated hemoglobin (%), mean (SD)	5.32 (0.23)	5.31 (0.23)	.43
FPG^b^ (mg/dL), mean (SD)	91.46 (4.99)	91.41 (5.01)	.74
1-hour PG (mg/dL), mean (SD)	129.47 (33.10)	128.86 (31.48)	.53
2-hour PG (mg/dL), mean (SD)	99.89 (18.41)	99.73 (18.34)	.77
SPG^c^ (mg/dL), mean (SD)	320.81 (45.05)	320.00 (43.52)	.54
Fasting IRI^d^ (IU/mL), mean (SD)	5.66 (3.13)	5.59 (2.71)	.47
1-hour IRI (IU/mL), mean (SD)	51.06 (33.02)	49.20 (31.85)	.06
2-hour IRI (IU/mL), mean (SD)	34.16 (22.88)	33.45 (21.68)	.28
S-IRI^e^ (IU/mL), mean (SD)	90.88 (51.66)	88.24 (49.38)	.08
ISI^f^ (composite), mean (SD)	9.93 (5.50)	9.98 (5.35)	.77
Systolic BP^g^ (mm Hg), mean (SD)	121.77 (16.63)	121.97 (16.01)	.68
Diastolic BP (mm Hg), mean (SD)	77.55 (10.82)	77.38 (10.51)	.59
Total cholesterol (mg/dL), mean (SD)	196.35 (30.13)	196.35 (30.28)	>.99
HDLC^h^ (mg/dL), mean (SD)	59.53 (14.30)	60.25 (15.01)	.11
LDLC^i^ (mg/dL), mean (SD)	114.88 (27.66)	113.94 (27.93)	.27
Triglyceride (mg/dL), mean (SD)	101.64 (63.13)	102.36 (77.61)	.75
Uric acid (mg/dL), mean (SD)	6.05 (1.28)	6.06 (1.31)	.70
UN^j^ (mg/dL), mean (SD)	13.35 (3.04)	13.43 (2.99)	.36
Serum creatinine (mg/dL), mean (SD)	0.87 (0.14)	0.87 (0.14)	.95
GOT^k^ (IU/L), mean (SD)	22.96 (8.20)	22.81 (8.42)	.55
GPT^l^ (IU/L), mean (SD)	23.12 (13.91)	22.72 (13.13)	.32
γ‐GTP^m^ (IU/L), mean (SD)	40.74 (45.54)	39.87 (46.02)	.53
Serum albumin (g/dL), mean (SD)	4.50 (0.26)	4.49 (0.27)	.47

^a^Used *t* test or chi-square test.

^b^FPG: fasting plasma glucose.

^c^SPG: sum of plasma glucose.

^d^IRI: immunoreactive insulin.

^e^S-IRI: sum of immunoreactive insulin.

^f^ISI: insulin sensitivity index.

^g^BP: blood pressure.

^h^HDLC: high-density lipoprotein cholesterol.

^i^LDLC: low-density lipoprotein cholesterol.

^j^UN: serum urea nitrogen.

^k^GOT: serum glutamic oxaloacetic transaminase.

^l^GPT: serum glutamic pyruvic transaminase.

^m^γ‐GTP: serum γ-glutamyl transpeptidase.

**Table 4:** List of trained classification models.

Model	Algorithm	Input variables
A	XGBoost	Sex, age, BMI^a^, HbA_1c_^b^, FPG^c^, and fasting IRI^d^
B	XGBoost	Variables in model A, 1-hour PG, 2-hour PG, 1-hour IRI, and 2-hour IRI
C	XGBoost	Variables in model B, SPG^e^ during the 75-g OGTT^f^, S-IRI^g^ during the OGTT, simple ISI^h^ (composite), systolic blood pressure, diastolic blood pressure, total cholesterol, HDLC^i^, LDLC^j^, triglyceride, uric acid, UN^k^, serum creatinine, GOT^l^, GPT^m^, γ‐GPT^n^, and serum albumin
D	LR^o^	FPG
E	LR	HbA_1c_
F	LR	FPG, HbA_1c_
G	LR	Sex, age, BMI, HbA_1c_, FPG, and fasting IRI
H	LR	Variables in model A, 1-hour PG, 2-hour PG, 1-hour IRI, and 2-hour IRI
I	LR	Variables in model B, SPG during the 75-g OGTT, S-IRI during the OGTT, simple ISI (composite), systolic blood pressure, diastolic blood pressure, total cholesterol, HDLC, LDLC, triglyceride, uric acid, UN, serum creatinine, GOT, GPT, γ‐GPT, and serum albumin

^a^BMI: body mass index.

^b^HbA_1c_: glycated hemoglobin.

^c^FPG: fasting plasma glucose.

^d^IRI: immunoreactive insulin.

^e^SPG: sum of plasma glucose.

^f^OGTT: oral glucose tolerance test.

^g^S-IRI: sum of immunoreactive insulin.

^h^ISI: insulin sensitivity index.

^i^HDLC: high-density lipoprotein cholesterol.

^j^LDLC: low-density lipoprotein cholesterol.

^k^UN: serum urea nitrogen.

^l^GOT: serum glutamic oxaloacetic transaminase.

^m^GPT: serum glutamic pyruvic transaminase.

^n^γ‐GPT: serum γ‐glutamyl transpeptidase.

^o^LR: logistic regression.

## Results

### Study 1. Prediction of Future Risk of Developing Diabetes

**Figure 3:**
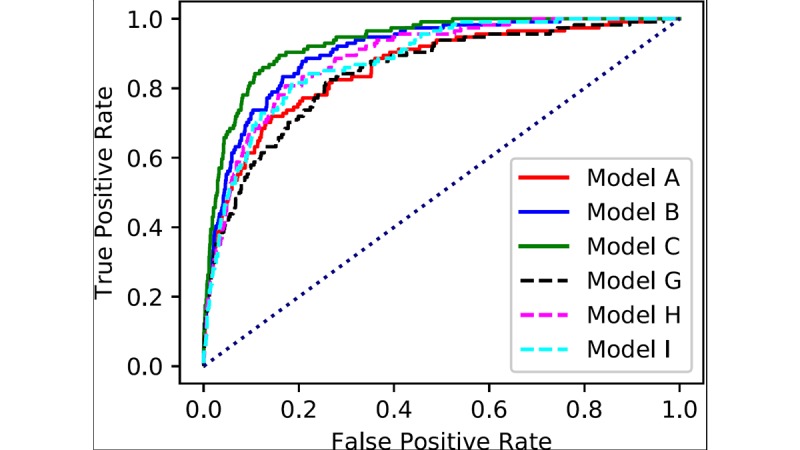
Receiver operating characteristic curves obtained for the prediction of diabetes.

Figure 3 shows the 6 ROC curves for models A to I. Similarly, Multimedia Appendix 2 shows the corresponding curves for models A to F. The horizontal axis represents the false positive rate, and the vertical axis represents the true positive rate. The 3 solid lines (models A, B, and C) show the ROC curves obtained from XGBoost. The dashed lines (models G, H, and I) show the ROC curves obtained from LR.

The AUC values for the 9 classification models are shown in Table 5. For each model, we also show the sensitivity and specificity as determined by the Youden index. We observed that XGBoost had superior performance compared with LR. The AUC value increased with the number of input variables. Models B and C, which exploit XGBoost and complete OGTT information for input variables, showed the best AUC values, 0.90 and 0.93, respectively.

In addition, XGBoost provides an importance score for each input variable. The importance value for each input variable in models A to C are shown in Multimedia Appendix 3 (left), Multimedia Appendix 4 (left), and Multimedia Appendix 5 (left), respectively. In model B, we observed that the OGTT variables (1-hour PG, 1-hour IRI, 2-hour PG, and 2-hour IRI) were more important than FPG or HbA_1c_. In model C, we observed that SPG and 2-hour PG were more important variables than FPG or HbA_1c_, although multicollinearity needs to be considered.

### Study 2. Prediction of Future Risk of Glucose Metabolism Disorders

**Figure 4:**
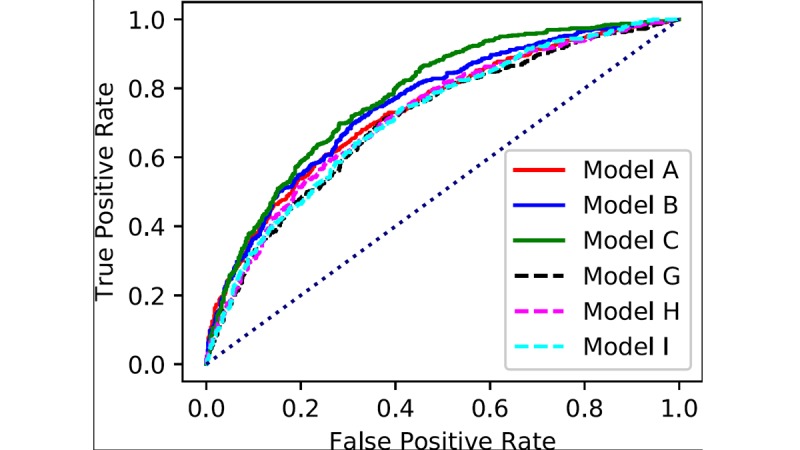
Receiver operating characteristic curves for the prediction of glucose metabolism disorders.

Figure 4 shows the 6 ROC curves for models A to I. Similarly, Multimedia Appendix 6 shows the corresponding 6 ROC curves for models A to F. The 3 solid lines (models A, B, and C) show the ROC curves obtained from XGBoost. The dashed lines (models G, H, and I) show the ROC curves obtained from LR.

**Table 5:** Area under the curve, sensitivity, and specificity for predicting diabetes.

Type and model		AUC^a^	Sensitivity (%)	Specificity (%)
**Machine learning**				
	Model A	0.86	36.8	97.2
	Model B	0.90	40.4	97.4
	Model C	0.93	39.5	98.4
**Logistic regression**				
	Model D	0.80	12.3	99.4
	Model E	0.78	6.1	99.9
	Model F	0.84	41.2	95.2
	Model G	0.85	38.6	95.9
	Model H	0.88	37.7	96.1
	Model I	0.88	40.4	96.5

^a^AUC: area under the curve.

The AUC values for the 9 models are shown in Table 6. The sensitivity and specificity, as determined by the Youden index, are also shown in Table 6. Similar to study 1, we observed that XGBoost had better performance than LR. The AUC values also increased with the number of input variables. Models B and C, which exploit XGBoost and complete OGTT information as input variables, displayed the highest AUC values, 0.75 and 0.78, respectively.

The importance score of each input variable for models A, B, and C are shown in Multimedia Appendix 3 (right), Multimedia Appendix 4 (right), and Multimedia Appendix 5 (right), respectively. In model B, we observed that the OGTT variables (1-hour PG, 1-hour IRI, 2-hour PG, and 1-hour IRI) were more important than FPG or HbA_1c_. In model C, we observed that the OGTT variables (1-hour PG, 1-hour IRI, 2-hour PG, 2-hour IRI, and SPG) were more important variables than FPG or HbA_1c_, although multicollinearity needs to be considered.

**Table 6:** Area under the curve, sensitivity, and specificity for predicting glucose metabolism disorders.

Type and model		AUC^a^	Sensitivity (%)	Specificity (%)
**Machine learning**				
	Model A	0.73	34.3	90.9
	Model B	0.75	31.6	91.8
	Model C	0.78	33.7	92.2
**Logistic regression**				
	Model D	0.65	6.6	97.3
	Model E	0.63	21.3	91.6
	Model F	0.69	6.6	98.4
	Model G	0.71	27.6	78.9
	Model H	0.72	27.1	91.6
	Model I	0.72	41.3	84.9

^a^AUC: area under the curve.

## Discussion

### Principal Findings

In this study, we reported on 2 results for predicting the future risk of diabetes or GMD using complete OGTT information and machine learning.

A feature of our study is that we used a large-scale dataset that included thousands of Japanese OGTT trials, even though OGTT is expensive and invasive. The amount of data enabled us to use a machine learning approach. It is known that one of the earliest detectable abnormalities in the development of diabetes is the deterioration of the early insulin response after glucose loading [28], and the aggravation of insulin sensitivity affects the development of diabetes [7,29-32]. Data obtained from OGTT provide important pathological glucose metabolism information. We believe that the data obtained from OGTT contributed to the improvement in prediction of future risk of diabetes and GMD.

Another feature of our study is that we used an advanced and powerful machine learning method, XGBoost, which resulted in better performance compared with LR. Many previous diabetes risk assessment tools used LR analyses. Recently, various machine learning algorithms have been used for the screening and prediction of diabetes [12,33-35]. Linear approaches are generally unsuited for prediction models with complex correlations. We believe that XGBoost plays an important role in improving the prediction of the future risk of developing diabetes or GMD. To our knowledge, no previous study combined large-scale Japanese OGTT data and XGBoost.

By observing the importance scores (Multimedia Appendix 3-Multimedia Appendix 5) of the input variables computed from XGboost, the OGTT variables (1-hour PG, 1-hour IRI, 2-hour PG, 2-hour IRI) were found to be more important predictors than FPG or HbA_1c_ for the future risk of diabetes or GMD, although multicollinearity needs to be taken into account. The progress of the PG level after loading can reflect abnormalities in the insulin response. Simultaneous measurement of PG and IRI levels can help evaluate the insulin sensitivity. Although multiple collinearity affects the results, PG and IRI levels after loading appeared to be more important than FPG or HbA_1c_.

### Limitations

Our research had several limitations. First, we did not use any information obtained from questionnaires in our research. This was because we were concerned that subject recall bias may impact the accuracy of the predictions [36]. As far as we know, previous diabetes risk assessment tools were based on information obtained from questionnaires such as family history and lifestyle habits [5,8-10,37]. Because of this, our results could not be easily compared with these reports. A previous study showed that combining the results of blood tests and questionnaire information improved the prediction accuracy of diabetes risk assessment [37]. We will attempt to improve the accuracy of diabetes risk prediction by integrating information obtained from blood tests and questionnaires. Second, we merged data from subjects who underwent OGTT different numbers of times. That is, we handled data from subjects who had 2 OGTT trials in the same manner as subjects with 10 OGTT trials. Data from subjects who had frequent OGTT trials may have impacted the calculation. Finally, our subjects were affected by selection bias, specifically, the “healthy worker” effect. More than 70% of our participants were healthy male office workers who ranged in age from 40 to 60 years. Thus, the limited sample might not accurately represent the entire population. In addition, we believe that our method is not suitable for predicting rapidly progressing diabetes, as with type 1 diabetes. Also, validation is still required using other data sets.

### Comparison With Prior Work

Thoopputra et al [5] considered many diabetes risk assessment tools developed worldwide. In the review, although there a few that used OGTT data or decision tree algorithms, many diabetes risk assessment tools used only noninvasive information and LR analyses. Values for the AUC ranged from 62% to 87%. In Japan, Nanri et al [37] reported a risk score showing an AUC value of 0.882 for predicting type 2 diabetes based on noninvasive information, FPG level and HbA_1c_, using an LR analysis. Recently, studies have demonstrated new methods, including machine learning algorithms, big data mining approaches, and genomic information, for the screening and prediction of diabetes [11,12]. Habibi et al [34] developed a model with an AUC value of 0.875 when screening for type 2 diabetes that used a decision tree method and did not require any laboratory tests. López et al [12] reported a model for diabetes prediction having an AUC value of 0.89 that used genetic information and a random forest algorithm.

### Conclusion

Our predictions for the future risk of developing diabetes or GMD, using data from thousands of OGTT trials and the machine learning program XGBoost, resulted in higher accuracy compared with traditional LR analysis. Combining complete OGTT information with advanced machine learning algorithms may be useful for detecting the future risk of diabetes or GMD more accurately.

## Appendix

Multimedia Appendix 1 Brief review of gradient tree boosting algorithms.

Multimedia Appendix 2 Receiver operating characteristic curves in study 1, comparison with reference models D-F.

Multimedia Appendix 3 Importance of variables in model A.

Multimedia Appendix 4 Importance of variables in model B.

Multimedia Appendix 5 Importance of variables in model C.

Multimedia Appendix 6 Receiver operating characteristic curves for study 2, comparisons with reference models D-F.

## Abbreviations

AUC: area under the curve
BMI: body mass index
BP: blood pressure
FPG: fasting plasma glucose
GMD: glucose metabolism disorders
GOT: serum glutamic oxaloacetic transaminase
GPT: serum glutamic pyruvic transaminase
GTB: gradient tree boosting
HbA_1c_: glycated hemoglobin
HDLC: high-density lipoprotein cholesterol
IRI: immunoreactive insulin
ISI: insulin sensitivity index
JDS: Japan Diabetes Society
LDLC: low-density lipoprotein cholesterol
LR: logistic regression
NGT: normal glucose tolerance
NTT: Nippon Telegraph and Telephone Corporation
OGTT: oral glucose tolerance test
PG: plasma glucose
ROC: receiver operating characteristic
S-IRI: sum of immunoreactive insulin
SPG: sum of plasma glucose
UN: serum urea nitrogen
γ‐GTP: serum γ-glutamyl transpeptidase
