# Supplementary material for: Prediction of Glucose Metabolism Disorder Risk Using a Machine Learning Algorithm: Pilot Study
Source: JMIR Diabetes. 2018 Nov 26;3(4):e10212. doi: 10.2196/10212 (PMC6288596; doi:10.2196/10212)
Supplement: Multimedia Appendix 1 [file diabetes_v3i4e10212_app1.pdf]

Appendix A

In this appendix, GTB algorithm is briefly reviewed according to Chen and Guestrin [16]. Let $x \in \mathbb{R}^m$ be m dimensional input variables, $y \in \mathbb{R}$ be an output variable, and $D = \{(x_i, y_i)_{i=1}^n | x_i \in \mathbb{R}^m, y_i \in \mathbb{R}\}$ be n number of data. A regression tree $f(x)$ having T leaves and leaf weight vector $w \in \mathbb{R}^T$ is defined as

$$f(x) = w_{q(x)},$$
$$q: \mathbb{R}^m \to \{1, \dots, T\}, w \in \mathbb{R}^T,$$

where q is a function that maps input variables $x \in \mathbb{R}^m$ to leaf index $i \in \{1, \dots, T\}$. The function q specifies the tree structure of a regression tree. $w \in \mathbb{R}^T$ specifies the leaf weights of the regression tree. Let $\mathcal{F}$ be the whole set of the regression trees having T leaves.

In ensemble learning such as Bagging and Boosting, outcome y is predicted using an ensemble $\phi(x)$ of K number of weak-learners $\{f_k(x)\}$:

$$\hat{y}_i = \phi(x_i) = \sum_{k=1}^{K} f_k(x_i),\ f_k \in \mathcal{F}$$

For each regression tree $f_k(x)$, we need to learn tree structure q and leaf weights $w \in \mathbb{R}^T$ from training data. Tree structure q and leaf weights $w \in \mathbb{R}^T$ are learned by minimizing the following regularized objective function:

$$L(\phi) = \sum_{i=1}^{n} l(y_i, \hat{y}_i) + \sum_{k=1}^{K} \Omega(f_k),$$

$$\Omega(f) = \gamma T + \frac{1}{2}\lambda \|w\|^2,$$

Here l is a differentiable convex function that measures difference between predicted values $\hat{y}_i$ and the true value y_i (fitting loss). $\Omega(f)$ is a regularization term introduced in order to prevent over-fitting. The penalty term γT is added so that leaf number T is small and resulting regression tree $f_k(x)$ is simple. The penalty term $\frac{1}{2}\lambda\|w\|^2$ is added so that $l2$-norm of the leaf weights w is small. γ, λ are hyperparameters of the XGBoost.

In the Gradient Boosting, a weak-learner f_k is trained in additive manner. Let $\hat{y}_i^{(t)}$ be the predicted value for the i-th data at iteration step t. Using a new function f_t, objective function $L(\phi)$ is rewritten as follows:

$$L^{(t)}(f_t) = \sum_{i=1}^{n} l\left(y_i\ , \hat{y}_i^{(t-1)} + f_t(x_i)\right) + \Omega(f_t).$$

Given $\hat{y}_i^{(t-1)}$, regression tree f_t is greedily optimized to minimize the objective function $L^{(t)}(f_t)$. XGBoost exploits the second order approximation of $L^{(t)}(f_t)$ using Taylor expansion, i.e.,

$$L^{(t)}(f_t) \approx \sum_{i=1}^{n} \left[l\left(y_i\ , \hat{y}_i^{(t-1)}\right) + g_i f_t(x_i) + \frac{1}{2} h_i f_t^2(x_i)\right] + \Omega(f_t).$$

Here $g_i = \partial_{\hat{y}_i^{(t-1)}} l\left(y_i\ , \hat{y}_i^{(t-1)}\right)$ and $h_i = \partial^2_{\hat{y}_i^{(t-1)}}\ l\left(y_i\ , \hat{y}_i^{(t-1)}\right)$ are gradient and hessian statistics computed from first and second order derivatives of the loss function l, respectively.

Objective function $L^{(t)}(f_t)$ as a function of f_t is simplified as

$$\tilde{L}^{(t)} = \sum_{i=1}^{n} \left[g_i f_t(x_i) + \frac{1}{2} h_i f_t^2(x_i)\right] + \gamma T + \frac{1}{2}\lambda \|w\|^2.$$

For each leaf $j \in \{1, \ldots, T\}$ of a regression tree f_t with a tree structure q, we define an index set $I_j = \{i | q(x_i) = j\}$. We define $G_j = \sum_{i \in I_j} g_i$, $H_j = \sum_{i \in I_j} h_i$.

Objective function $\tilde{L}^{(t)}$ is rewritten as

$$\tilde{L}^{(t)} = \sum_{j=1}^{T} \left[G_j w_j + \frac{1}{2}(H_j + \lambda) w_j^2\right] + \gamma T.$$

Note that the summation is only taken through leaves $j \in \{1, \ldots, T\}$ and not through data. Since the objective function $\tilde{L}^{(t)}$ is quadratic with respect to leaf weight vector w, the optimal leaf weights can be solved as

$$w_j^* = -\frac{G_j}{H_j + \lambda}\ j \in \{1, \ldots, T\}.$$

By substituting the optimal weight vector w^* into the objective function $\tilde{L}^{(t)}$, we obtain

$$\tilde{L}^{(t)}(q) = -\frac{1}{2} \sum_{j=1}^{T} \frac{G_j^2}{H_j + \lambda} + \gamma T$$

Note that once a tree structure q of a regression tree f_t is given, the optimal weight vector w^* and $\tilde{L}^{(t)}(q)$ are computed. Objective function $\tilde{L}^{(t)}(q)$ is used as a score of the tree structure q.

It is computationally severe to enumerate all candidates of tree structures q. To

obtain an efficient and approximate algorithm, a greedily optimal splitting of a leaf node is explored using the score function $\tilde{L}^{(t)}(q)$. Let q_B be a tree structure before splitting of a leaf node and q_A be a tree structure after splitting of the leaf node. Then the gain of the splitting is measured by a score:

$$L_{split} = \tilde{L}^{(t)}(q_B) - \tilde{L}^{(t)}(q_A).$$

A greedily optimal splitting is determined by that maximizing the gain. A greedily optimal tree structure q^* is explored by repeating the above procedure. XGBoost is elaborated on speeding up the above algorithm and increasing the scalability, with taking into account sparsity-aware algorithm, out-of-core computing, and parallel computing. See Chen and Guestrin [16] for technical details.
