# Supplementary figures and images for: Prediction of Glucose Metabolism Disorder Risk Using a Machine Learning Algorithm: Pilot Study
Source: JMIR Diabetes. 2018 Nov 26;3(4):e10212. doi: 10.2196/10212 (PMC6288596; doi:10.2196/10212)

## Slide 1
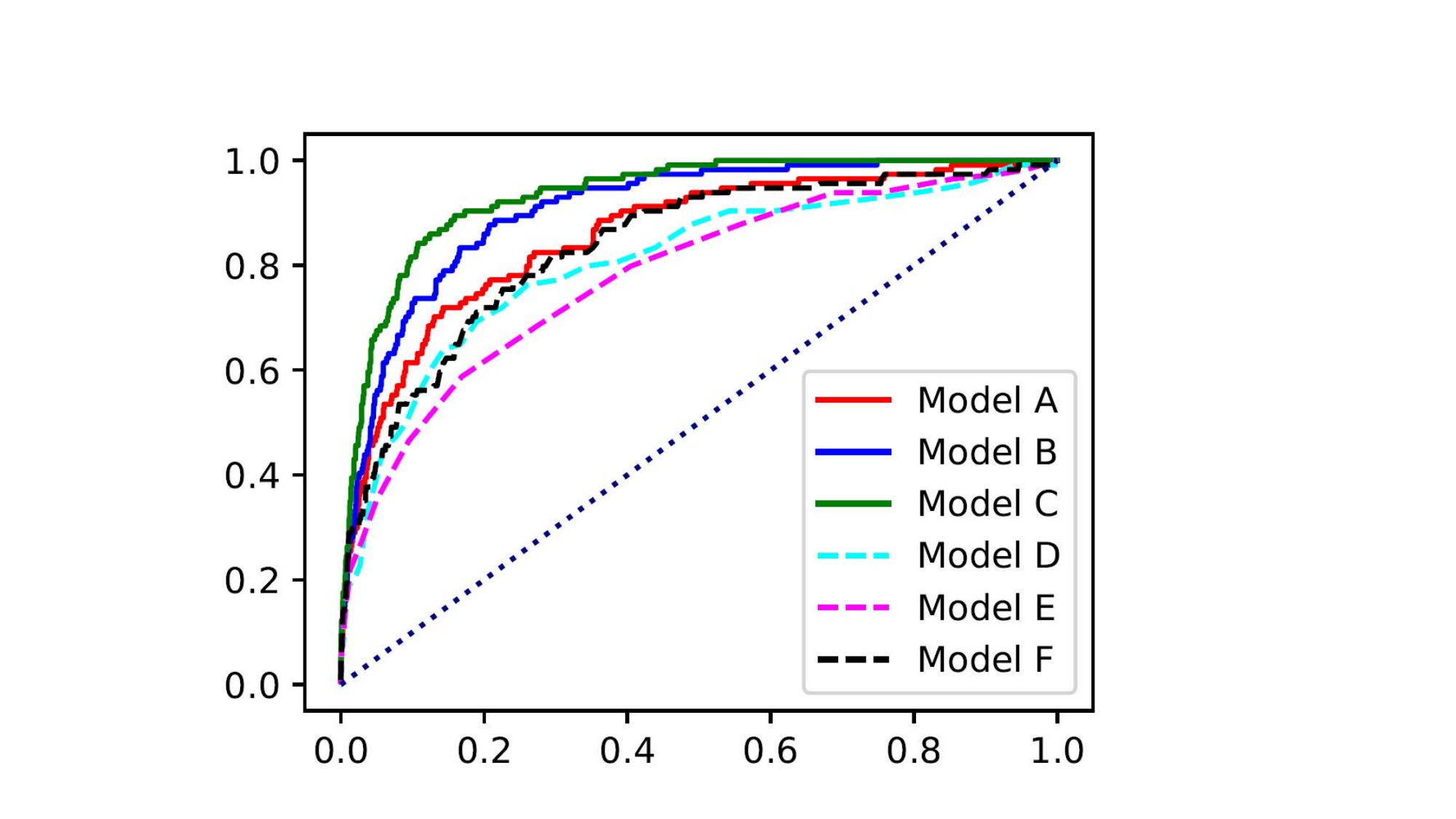

Supplement: Multimedia Appendix 2 [file diabetes_v3i4e10212_app2.pptx]

## Slide 1
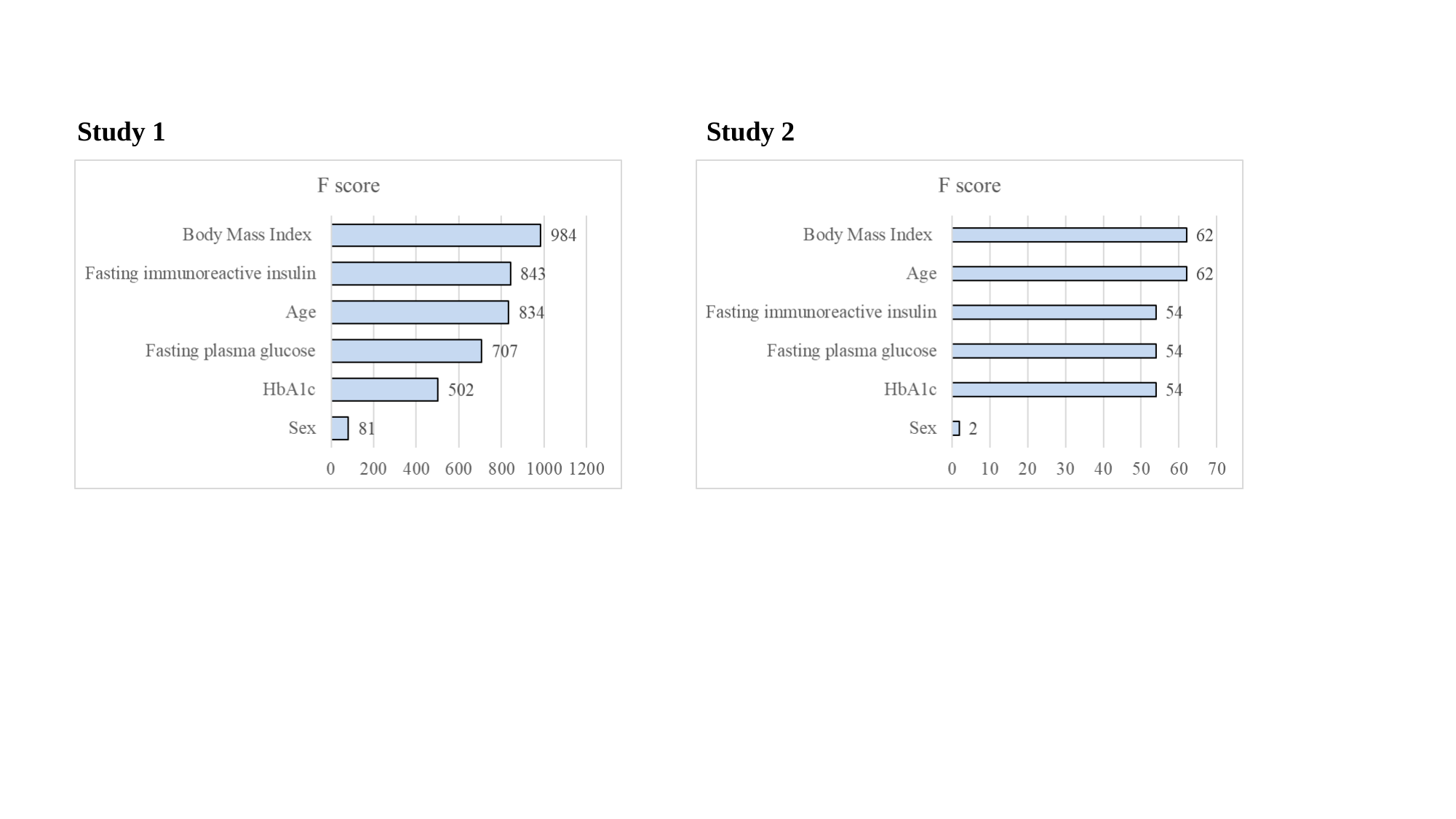

Study 1
Study 2

Supplement: Multimedia Appendix 3 [file diabetes_v3i4e10212_app3.pptx]

## Slide 1
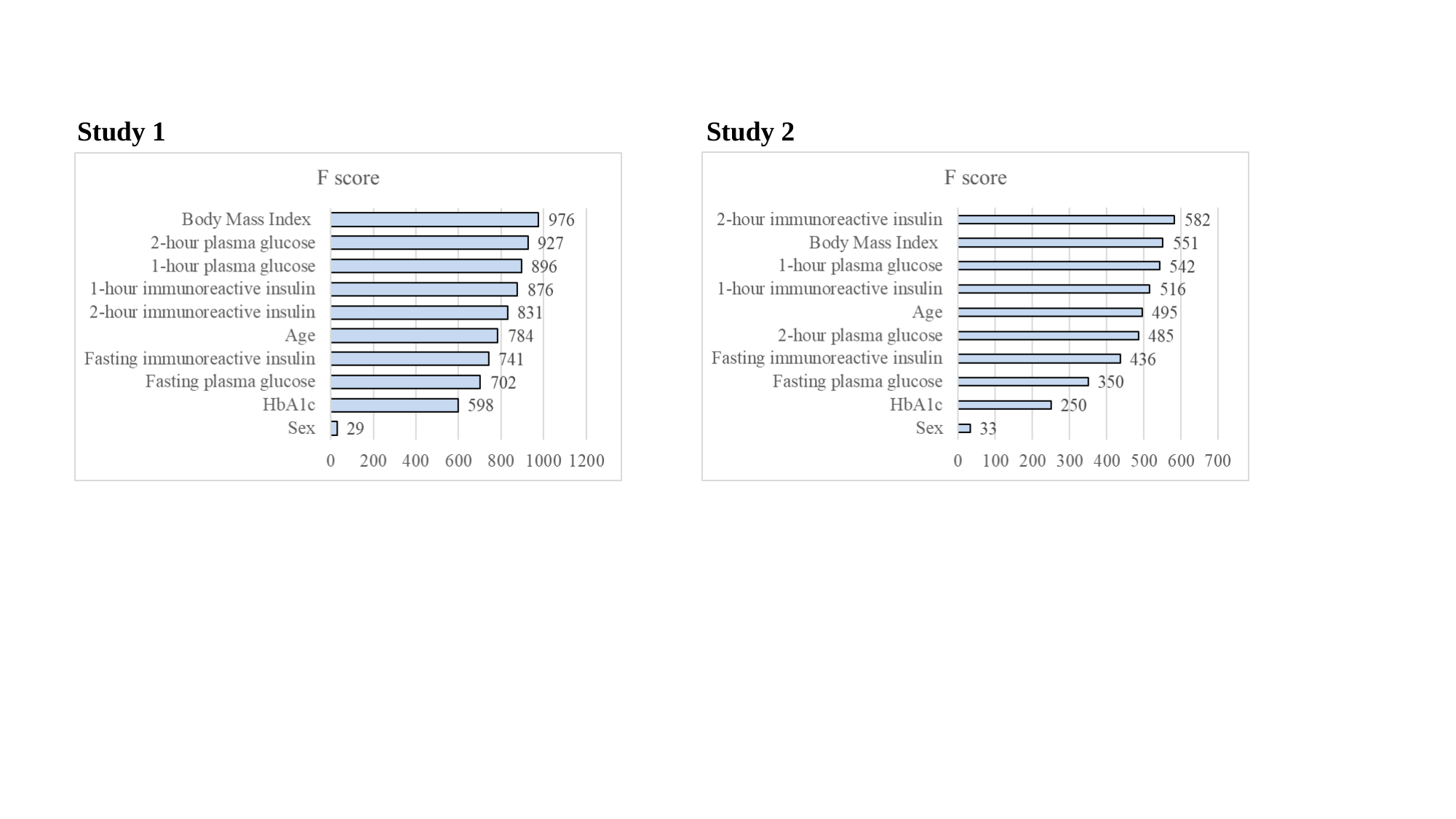

Study 1
Study 2

Supplement: Multimedia Appendix 4 [file diabetes_v3i4e10212_app4.pptx]

## Slide 1
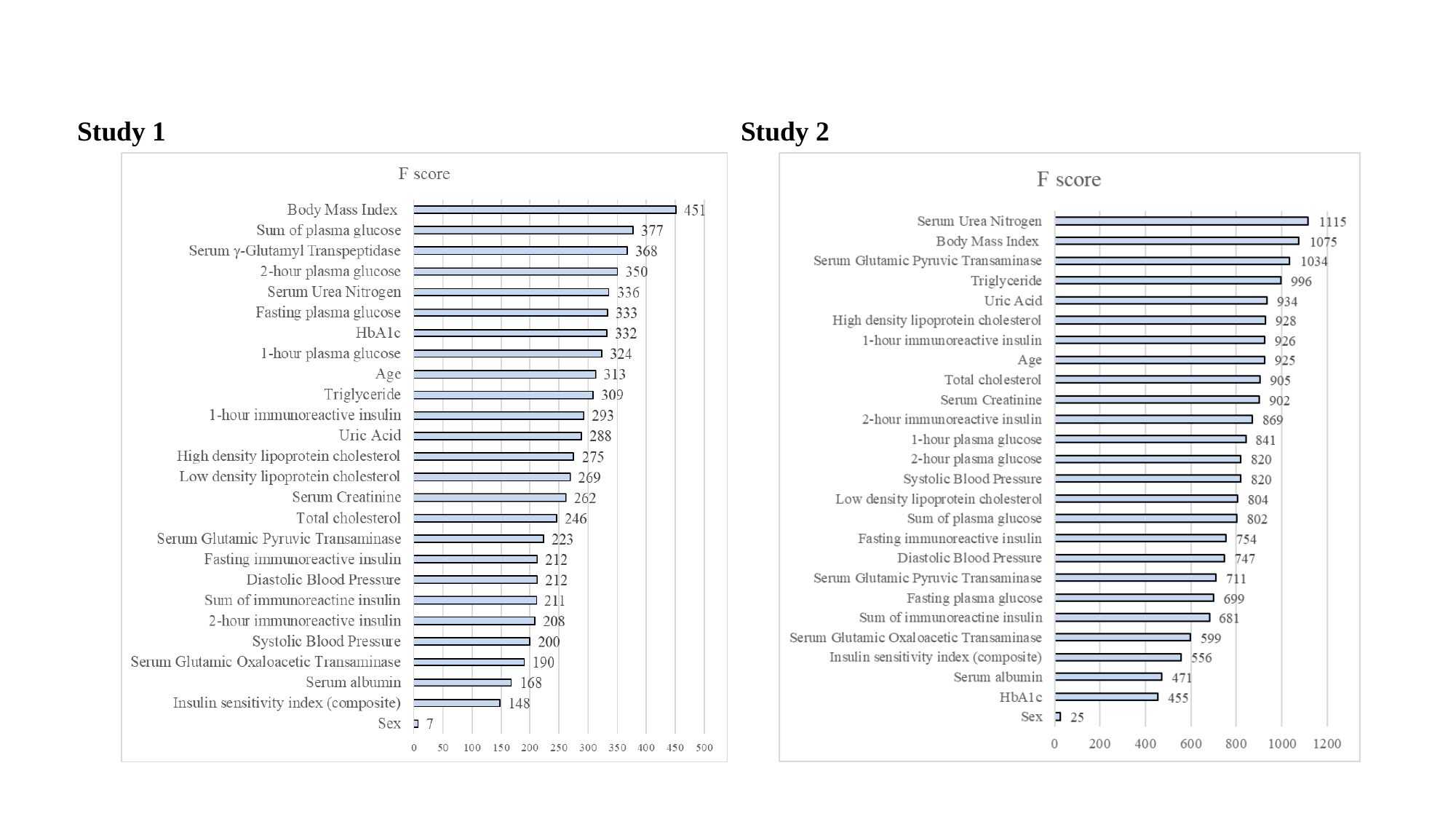

Study 1
Study 2

Supplement: Multimedia Appendix 5 [file diabetes_v3i4e10212_app5.pptx]

## Slide 1
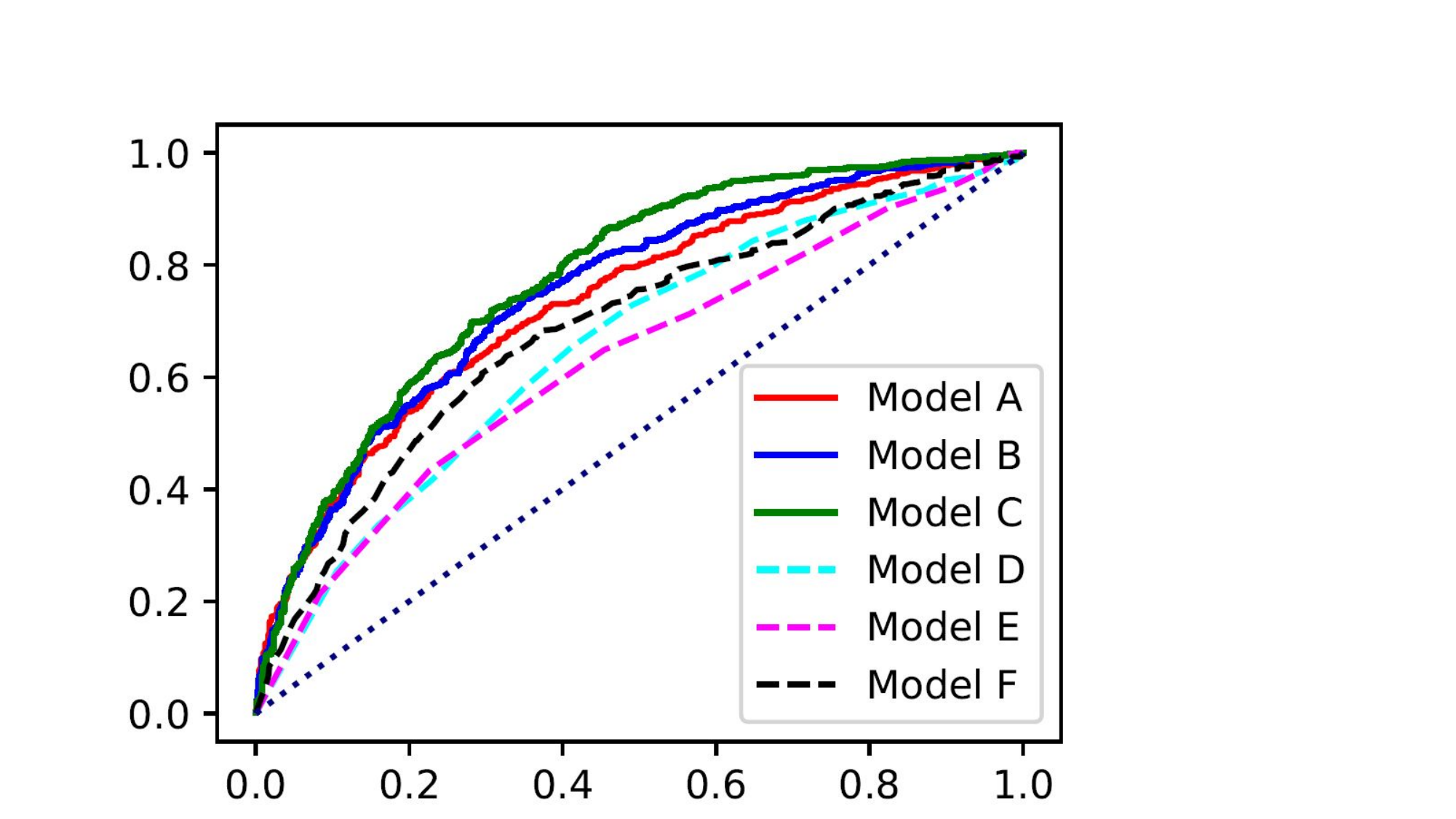

Supplement: Multimedia Appendix 6 [file diabetes_v3i4e10212_app6.pptx]
